# To what extent do young chinese elites comply with COVID-19 prevention and control measures?

**DOI:** 10.1186/s12889-023-15643-6

**Published:** 2023-04-24

**Authors:** Yuanyuan Huang, Hua Zhang, Zixuan Peng, Min Fang

**Affiliations:** 1 School of Political Science and Public Administration, Guangxi Minzu University, Nanning, Guangxi China; 2 Dalla Lana School of Public Health, University of Toronto, 155 College St, 4th Floor, Toronto, Ontario M5T 3M6 Canada; 3 School of Public Administration, South China Agriculture University, Guangzhou, China

**Keywords:** COVID-19, Prevention and control policies, Young elites, Compliance behaviour

## Abstract

**Background:**

During the COVID-19 pandemic, it is vital for individuals to comply with the government’s prevention and control measures. This study aims to explore determinants of college students’ compliance behaviour during the COVID-19 pandemic.

**Methods:**

This study conducted an online survey among 3,122 individuals aged 18 and above from March to November 2022 in China. Individuals’ compliance behaviour was divided into protective behaviour (that includes wearing a mask, maintaining a physical distance, and getting vaccinated) and restrictive behaviour (that includes offering health codes and a nucleic acid test certificate). Individuals’ compliance motivation was divided into calculated motivation (including the fear of being infected, the fear of being published, and previous experience of pandemic prevention) and normative motivation (including the sense of social responsibility and trust in government). We defined young people aged between 18 and 24 with a college degree as young elites, and constructed ordinary least squares linear regression to compare their compliance behaviour with young people without a college degree (young non-elites), and non-young people with a college degree (non-young elites).

**Results:**

Almost three years after the outbreak of the pandemic, Chinese individuals retained a high degree of compliance with COVID-19 prevention and control policies, particularly with respect to the provision of health codes. Young elites were more compliant with getting vaccinated, wearing a mask, providing health codes and testing results than their counterparts. The sense of social responsibility and trust in government were the major drivers of young elites’ compliance behaviour during the pandemic. Young elites who were male, had a rural “hukou”, and were not a member of the China Communist Party were more compliant with COVID-19 prevention and control measures.

**Conclusion:**

This study found that young elites in China had high policy compliance during the COVID-19 pandemic. These young elites’ compliance behaviour was driven by their sense of social responsibility and trust in government rather than the fear of being infected and the fear of being punished as a result of violating the regulations. We suggest that in the context of managing health crises, in stead of introducing punitive measures to enforce citizens to comply with the management measures, promoting citizens’ sense of social responsibility and building a trusting relationship with citizens contrite to the enhancement of citizens’ policy compliance.

## Background

Since the outbreak of the COVID-19 pandemic, the Chinese government introduced and implemented a set of strict prevention and control policies. Alongside the vaccination programs, mask-wearing, social distancing, home isolation, and quarantine measures, the Chinese government also implemented compulsory nucleic acid-based testing and a requirement for people to show electronic health records of their health status when entering and leaving public places. These measures have been in place for a long period until the Chinese government loosen the strict control policies in early December 2022. Policy compliance refers to citizens’ willingness to comply with policies, the positive actions they take to cooperate with and implement these policies, and their demonstration of attitudes and behaviour that support the achievement of the policy objectives [1–2]. Plenty of studies have suggested that citizens’ compliance to public health policies is of paramount significance to contain the spread of virus during a pandemic [3–9]. However, it remains a challenge for the governments to boost citizens’ willingness to comply with the prevention and control policies [4]. Particularly, citizens’ degree of compliance to public health interventions may decline over time as a result of maintaining a high level of self-discipline for a long period of time [10–12]. As such, it is of essential significance to examine whether the prevention and control policies remained recognised and adhered to by the public nearly three years after the outbreak of COVID-19 in China and before they were recently abolished.

To do so, the study focused on college students in China, who have three important characteristics that may greatly influence their compliance behaviour. First, college students are an important source of economic, intellectual and political elites [13, 14]. Second, college students are young and have little social experience. Although they constitute the younger generation of elites, Sunstein [15] found that young people tend to be more rebellious and less likely to comply with policies introduced by the government, or may also be more compliant with the policies [16]. Third, most students live in a centralised campus in the universities with large populations and frequent contact, and most universities in China implemented closed management. However, it also means that many students were thus placed in high pressure situations, with classes suspended and strict isolation measures imposed. In following the pandemic prevention policies, such as restrictions on personal freedom, students experienced great inconvenience and stress, with limited external communication. This increased their negative emotions, such as anxiety, impatience, fear and depression, potentially leading to mental health problems and it has brought great challenges to social stability [17–19].

Previous studies have found some contradictory conclusions, particularly for age and education. Some studies have found that higher education levels resulted in higher levels of compliance with policies of handwashing and wearing masks [16, 20, 21]. In contrast, others found that intellectual elites evaded policies that they regarded as unfavourable [22] and found that education attainment did not affect citizens’ compliance with keeping social distancing during the COVID-19 pandemic [23]. Likewise, some studies have shown that young people were more rebellious and less likely to comply with policies introduced by the government than their older counterparts [15, 20], while the opposite findings have been found in other research [16].

Most of prior studies analyzed individuals’ motivation for their compliance behaviour from two perspectives, referred to as the calculated motivation and the normative motivation. ***The calculated motivation theory*** is based on the view that people rationally analyzed the rewards and costs of their compliance behaviour [24], which specifically contains the deterrence theory, protection motivation theory, and prospect theory. ***The deterrence theory*** advocates that non-compliant behaviour of citizens ought to be controlled and restrained by designing policies with considerable violation costs [25]. Under this theory, the strict and compulsory enforcement of policies increased citizens’ motivation to comply with policies [26]. Some studies have found that the greater the threat of punishment, the less likely college student participated in illegal computer activities [27]. However, other studies have shown that excessive sanctions not only increased the cost of compliance but also drastically reduced the enthusiasm of citizens for voluntary compliance and destroyed the trust relationship between the government and citizens [28]. ***The protection motivation theory*** focuses on individuals’ psychological motivation and claims that individuals made decisions based on their perception of risks and self-efficacy [29–31]. Specifically, studies have shown that citizens tended to comply with containment measures during the COVID-19 epidemic when they perceived a high level of risks [32–34] and were confident in following the introduced measures [35, 36]. Research on college students in Wuhan and Korean has shown that high risk awareness can promote preventive behaviour among college students with respect to the influenza A (H1N1) or COVID-19 epidemic [10, 37]. Nevertheless, other studies have demonstrated that a high level of perceived risks was associated with a sense of hopefulessness that reduced citizens’ compliance with policies [38]. ***The prospect theory*** holds that before making decisions in emergencies, individuals often dynamically adjusted their psychological reference points based on the situations of and actual losses associated with the emergencies [39, 40]. Hence, individuals were inspired by representative events and influenced by anchoring effects to adjust their behaviour in line with revised estimates of the likely costs and benefits of performing the behaviour.

***The calculated motivation theory***, however, is unable to offer a complete picture describing citizens’ compliance behaviour [31, 41]. The normative motivation theory suggests that individuals’ compliance behaviour was motivated by their intrinsic values that relate to their acceptance and recognition on government’s credibility, policy legitimacy, and policy fairness [42–46]. Some studies have shown that the sense of social responsibility plays an essential role in motivating individuals’ policy compliance behaviour during the COVID-19 pandemic [9, 47]. A positive and significant relationship has also been found between trust in government and college student’s compliance with taking preventive measures [3]. Improving college students’ public awareness and moral sensibilities can enhance their sense of responsibility and thus promote their participation in environmental protection [46].

In addition to risk perception [32] self-efficacy [48], and trust in government [33], and the sense of social responsibility [9], social interactions [24], pressure of social norms and peer pressure [49], emotion [50, 51], authoritarian control and risk communication [4] were also drivers of citizens’ compliance behaviour. Moreover, prior studies have explored the impacts of citizens’ demographic characteristics (such as age and education) on their compliance behaviour [37, 52, 53], although the evidence on the impacts of these demographic factors remain controversial.

Taken together, we examined the policy compliance behaviour of Chinese individuals, with the goal of offering valuable insights for China and other countries seeking to formulate interventions to deal with the health crisis through encouraging citizens to engage actively in pandemic management. Particularly, college students (hereafter referred to as young elites) are an important source of economic, intellectual and political elites who can exert a more substantial influence on the development of the society [13, 14, 54]. Meanwhile, these young elites frequently participated in social activities [55] and were subject to a range of COVID-19 prevention and control measures [56].

Many studies have examined the compliance behaviour of different age demographics (e.g., teenagers, college students and the elderly) during the COVID-19 pandemic [37, 52, 53]. However, no consistent conclusions have been reached regarding the influence of age and education level on compliance behaviour during a pandemic. While China has implemented several special measures, such as compulsory nucleic acid testing and health codes, these remain insufficient to analyse and discuss many compliance behaviours and their corresponding reasons in detail. For these reasons, our study took a particular interest on exploring young elites’ compliance behaviour. Therefore, our study focused on three issues. First, as young elites, what is the compliance level of college students with epidemic prevention policies? Second, what are the motivations for compliance? Third, what are the effects of educational advantages and age characteristics on individual compliance behaviours?

## Methods

### Data source

Travel restriction and social distancing were still in place at the time of carrying out this study, which prevented us from conducting a research in person. Thus, we distributed an online questionnaire to individuals aged 18 years and above from mid-March to mid-May (which yielded 2,364 questionnaires), from 15 August to late August (which yielded 583 questionnaires), and from early November to 30 November (which yielded 1,205 questionnaires) in 2022 across 29 administrative regions in China. We used snowball sampling, whereby the researchers distributed the questionnaire to individuals on a social media application (WeChat) and then these selected participants forwarded the questionnaires to other individuals satisfying the inclusion criteria. After excluding invalid samples, we finally obtained an analysis sample comprising 3,122 Chinese individuals.

The questionnaire was designed to explore individuals’ compliance behaviour and the underlying motivation for their compliance behaviour. The first section of the questionnaire included a brief introduction about the study objectives and procedures as well as a declaration to protect anonymity and confidentiality of participants. After being informed of the purposes of the research, all of the participants volunteered to participate in the survey. To ensure that the participants did not miss any questions, they had to answer all questions on each page before proceeding to the next page. We used previously validated scales to measure the variables of our interest, anonymized participants, and conducted exploratory factor analysis to reduce social desirability bias and ensure the consistency within the scales used.

### Standard protocol approvals, registrations, and participants consent

Informed consent was obtained from all the participants included in the study. This study was approved by the Academic Ethics Committee of School of Politics Science and Public Administration, Guangxi Minzu University (protocol number: 202,210,608,006). The present study was conducted in accordance with the principles of the 2013 Declaration of Helsinki.

### Variable measures

Individuals’ compliance behaviour was the outcome variable of interest. Individuals’ compliance behaviour was measured by asking ‘How often do you comply with the following prevention and control policies during the COVID-19 pandemic?’. The responses include wearing a mask, maintaining a physical distance of one meter from others in public places, getting vaccinated, providing a nucleic acid test certificate (NATC), and providing health codes or travel cards as a proof of health status (HCTC). A 4-point Likert scale [16] was used to rate each response (with 1 indicating ‘never’, 2 ‘occasionally’, 3 ‘sometimes’, and 4 ‘always’). The higher the score, the higher the degree was the participants’ compliance with COVID-19 prevention and control policies. The Cronbach’s α was more than 0.60 for the scale used to measure participants’ compliance behaviour. In addition, the questionnaire’s validity test showed that the P value of Bartlett test was less than 0.001 and the KMO value was 0.765, indicating that the scale had a good reliability and validity.

The result of exploratory factor analysis shows that there are two factors with variance explanation rates of 1.178 and 1.127 after orthogonal rotation. According to the nature of the policies promulgated in China and the results of factor analysis, we divided compliance behaviour into two categories. Factor 1 was named “protective behaviours” referred to the protective measures taken by respondents to protect themselves from infection in the face of the public health crisis. The corresponding behaviours included wearing a mask, maintaining a social distance of 1 m in public places and vaccinating, with factor loadings of 0.5348, 0.5852, and 0.4162, respectively. Factor 2 was named “restrictive behaviours “, which aimed to ensure the safety of others by restricting residents’ access to public places. Corresponding behaviours included providing NATC and HCTC, with factor loadings of 0.5278 and 0.5886. In the part of statistical description, we described five types of compliance behaviour. We used protective and restrictive behaviours as dependent variables in our regression analyses.

Individuals’ motivation for compliance was included as the key independent variable. Individuals’ motivation for compliance was measured by answering the question ‘Why are you willing to comply with the prevention and control policies during the COVID-19 pandemic? [9, 52]. The responses include 1) the fear of being punished (derived from the deterrence theory), 2) the fear of being infected and infecting family members and friends (derived from the protection motivation theory), 3) the previous experience of pandemic prevention (derived from the prospect theory), 4) the sense of social responsibility (derived from the normative motivation theory), and 5) trust in the government (derived from the normative motivation theory).

We also included age (young people aged 18–24 years or non-young people aged more than 24 years [57], education (whether the individual had a college degree or above), gender (male or female), “hukou” registration status (urban or rural), and political party membership (whether the participants was a member of the China Communist Party) as control variables. We classified our research participants into young elites (young people with a college degree or above), young non-elites (young people without a college degree or above), and non-young elites (non-young people with a college degree or above).

### Statistical methods

We used Stata 15.0 to conduct all the statistical analyzes and used a confidence level of 90%. We first conducted a descriptive analysis to explore individuals’ degree of compliance and motivation for their compliance with the prevention and control policies. Subsequently, we used the F-test and the chi-square test to compare young elites’ compliance degree and motivation for their compliance behaviour with other groups (including non-young elites and young non-elites). Finally, we used ordinary least squares linear regression to estimate the impacts of the proposed determinants of individuals’ protective and restrictive compliance behaviours.

## Results

### Results of descriptive analysis

Table 1 shows the descriptive characteristics of all the sampled individuals from 29 different administrative regions in China. Table 2 shows the average compliance level among young elites, non-young elites, and young non-elites. We found statistically significant differences in individuals’ compliance with wearing a mask (p = 0.06), keeping a physical distance (p < 0.001), getting vaccinated (p < 0.001), providing NATC (p < 0.001), and providing HCTC (p < 0.001) across the three groups. Young elites exhibited a high degree of compliance with providing HCTC (mean = 3.98), followed by getting vaccinated (mean = 3.94), providing NATC (mean = 3.91), wearing a mask (mean = 3.88), and maintaining a physical distance (mean = 3.56). Young elites had a higher level of compliance with all the prevention and control measures (that include wearing a mask, providing NATC, providing HCTC, and getting vaccinated) except for maintaining a physical distance than the other two groups.

**Table 1:** Descriptive statistics of the sampled participants

Variables	Description	Number (%)	
Motivation for compliance			
*Calculated motivation*	The fear of being punished	1125 (34.0)	
	The fear of being infected and infecting family members and friends	2423 (77.6)	
	The previous experience of pandemic prevention	2201 (70.5)	
*Normative motivation*	It is everyone’s responsibility and obligation to participate in pandemic control	2525 (80.9)	
	Trust in the government	2049 (65.6)	
Gender			
*Male*		1060 (34.0)	
*Female*		2062 (66.0)	
Age			
*Young people*	Individuals aged 18–24 years old	2261 (72.4)	
*Non-young people*	Individuals aged more than 24 years old	861 (27.6)	
Education			
*Elites*	Individuals with a college degree or above	2793 (89.5)	
*Non-elites*	Individuals without a college degree or above	329 (10.5)	
Age *Education			
*Young elites*	Young people with a college degree or above	1932 (61.9)	
*Yong non-elites*	Young people without a college degree	329 (10.6)	
*Non-young elites*	Non-young people with a college degree or above	861 (27.6)	
Hukou			
*Urban*		1444 (46.3)	
*Rural*		1678 (53.7)	
Political identity			
*China Communist Party member*		601 (19.3)	
*Non-party member*		2521 (80.7)	

**Table 2:** Comparisons of compliance behaviour among young elites, young non-elites, and non-young elites

	Wear a mask	Maintain distance	Vaccination	Provide NATC	Provide HCTC
	Mean (SD)	Mean (SD)	Mean (SD)	Mean (SD)	Mean (SD)
Young elites	3.88 (0.39)	3.56 (0.67)	3.94 (0.31)	3.91 (0.40)	3.98 (0.18)
Non-Young elites	3.83 (0.46)	3.40 (0.75)	3.83 (0.55)	3.74 (0.64)	3.93 (0.31)
Young non-elites	3.88 (0.45)	3.62 (0.69)	3.91 (0.41)	3.91 (0.42)	3.94 (0.29)
F statistic	5.07	19.80	21.84	38.93	13.37
p-value	0.063*	< 0.001***	< 0.001***	< 0.001***	< 0.001***

Note: *, ** and *** indicate p < 0.1, p < 0.05 and p < 0.01, respectively

Table 3 reports differences in young elites’ compliance behaviour across groups by gender, “hukou”, and political membership. Male young elites were substantially more compliant with maintaining a physical distance than their female counterparts (p < 0.001). Female young elites were more compliant with vaccinating (p = 0.04) and providing HCTC (p = 0.09) than their male counterparts. The young elites with a rural “hukou” were more compliant with wearing a mask (p < 0.001), maintaining a physical distance (p < 0.001), vaccinating (p = 0.10), and providing HCTC (p = 0.08) than those with a urban “hukou”. The non-party members were more compliant with maintaining a physical distance (p < 0.001) and providing NATC (p = 0.01) than those who were a member of the China Communist Party.

**Table 3:** Comparisons of compliance behaviour among young elites by gender, “hukou”, and political identity

Compliance behaviour	Gender			Hukou			Political membership		
	male	female	p-value	urban	rural	p-value	CCP member	party member	p-value
Wear a mask	3.87	3.89	0.202	3.84	3.91	< 0.001***	3.87	3.86	0.326
Maintain distance	3.65	3.51	< 0.001***	3.49	3.60	< 0.001***	3.58	3.39	< 0.001***
Vaccination	3.92	3.95	0.041**	3.92	3.95	0.097*	3.94	3.95	0.744
Provide NATC	3.91	3.91	0.802	3.90	3.91	0.424	3.92	3.84	0.007***
Provide HCTC	3.97	3.98	0.091*	3.97	3.98	0.078*	3.98	3.96	0.172

Note: *, ** and *** indicate p < 0.1, p < 0.05 and p < 0.01, respectively

### Comparisons of motivation for policy compliance

Table 4 shows the young elites’ motivation for their compliance behaviour. The most common reason that motivates young elites to comply was the sense of social responsibility (84.2%), followed by the fear of being infected and infecting family members and friends (78.1%). More than 70% of young elites complied because of their previous experience of pandemic prevention (73.2%) and trust in the government (71.3%). Less than half of the young elites complied with the prevention and control policies because of the fear of being punished if they rejected to comply with those policies (45.1%). These finding suggest that during the COVID-19 pandemic, the normative motivation and the calculated protection motivation promoted individuals’ policy compliance, while the deterrence motivation played a less important role in enhancing young elites’ policy compliance.

**Table 4:** Comparisons of motivations for young elites’ compliance behaviour

Type	Motivations	Yes -n (%)	No -n (%)
Calculated motivation	The fear of being punished	871 (45.1)	1061 (54.9)
	The fear of being infected and infecting family members and friends	1509 (78.1)	423 (21.9)
	The previous experience of pandemic prevention	1415 (73.2)	517 (26.8)
Normative motivation	The sense of social responsibility	1626 (84.2)	306 (15.8)
	Trust in government	1377 (71.3)	555 (28.7)

Table 5 reports comparisons of motivations for compliance behaviour between young elites and non-young elites. Young elites were more likely to comply because of the fear of being punished (p < 0.001), their previous experience (p < 0.001), trust in the government (p < 0.001) and the sense of social responsibility (p < 0.001) than non-young elites. This finding suggests that the normative motivation and the calculated motivation had greater impacts on young elites’ compliance behaviour than on non-young elites’. Table 6 reports comparisons of motivations for compliance behaviour between young elites and young non-elites. We found that the proportion of young elites who complied because of trust in government was slightly lower than that of young non-elites (p = 0.06).

**Table 5:** Comparisons of motivation for compliance behaviour between young elites and non-young elites

Type	Motivations	Young elites (%)	Non-young elites (%)	Chi^2^-value (P)
Calculated motivation	The fear of being punished	871 (45.1)	103 (12.0)	287.653 (< 0.001***)
	The fear of being infected and infecting family members and friends	1509 (78.1)	654 (76.0)	1.572 (0.210)
	The previous experience of pandemic prevention	1415 (73.2)	549 (63.8)	25.629 (< 0.001***)
Normative motivation	The sense of social responsibility	1626 (84.2)	624 (72.5)	51.946 (< 0.001***)
	Trust in government	1377 (71.3)	421 (48.9)	130.035 (< 0.001***)

Note: *, ** and *** indicate p < 0.1, p < 0.05 and p < 0.01, respectively

**Table 6:** Comparisons of motivation for compliance behaviour between young elites and young non-elites

Type	Motivations	Young elites (%)	Young non-elites (%)	Chi^2^-value(P)
Calculated motivation	The fear of being punished	871(45.1)	151(45.9)	0.075(0.9784)
	The fear of being infected and infecting family members and friends	1509(78.1)	260(79.0)	0.140(0.708)
	The previous experience of pandemic prevention	1415(73.2)	237(72.0)	0.207(0.649)
Normative motivation	The sense of social responsibility	1626(84.2)	275(83.6)	0.069(0.792)
	Trust in government	1377(71.3)	251(76.3)	3.512(0.061*)

Note: *, ** and *** indicate p < 0.1, p < 0.05 and p < 0.01, respectively

### The impacts of the proposed determinants on policy compliance

Table 7 shows the impacts of the proposed determinants of individuals’ compliance behaviour using the full sample. We found that individuals with a rural “hukou” (p < 0.001), were not a member of the China Communist Party (p = 0.04), were young (age ≤ 24) tended to comply with protective behaviours than their counterparts. Individuals’ previous experience (p = 0.003), the sense of social responsibility (p < 0.001) and trust in government (p < 0.001) increased individuals’ compliance with protective behaviour, while the fear of being published led to lower degrees of compliance with protective behaviour (p < 0.001). We also found that young people were more like to comply with restrictive behaviour (p < 0.001). The sense of social responsibility (p < 0.001) and trust in government (p < 0.001) were positively associated with individuals’ compliance with restrictive behaviour.

**Table 7:** The impacts of the proposed determinants on compliance behaviour using the full sample

*Variables*	*Model 1*	*Model 2*
	*Protective behaviour*	*Restrictive behaviour*
	*Coefficient (p-value)*	*Coefficient (p-value)*
Gender (Female)	-0.017	-0.001
Hukou (Rural)	0.057 ***	0.018
Political identity (CCP membership)	-0.038 **	-0.004
Young (aged ≤ 24)	0.065 ***	0.083 ***
Education (a college degree and above)	0.003	0.022
The fear of being punished	-0.072 ***	-0.012
The fear of being infected and infecting family members and friends	-0.015	0.021
The previous experience of pandemic prevention	0.046 ***	0.016
The sense of social responsibility	0.078 ***	0.057 ***
Trust in government	0.109 ***	0.065 ***
Constant	3.574 ***	3.712 ***
Number of observations	3122	3122
Adj. R-squared	0.054	0.041

Note: *, ** and *** indicate p < 0.1, p < 0.05 and p < 0.01, respectively

Table 8 reports the impacts of the proposed determinants of compliance behaviour using subsamples. We demonstrated that young elites who were male, had a rural “hukou”, and were not a member of the China Communist Party were more compliant with protective behaviour than their counterparts (p < 0.05). In comparison, young non-elites with a rural “hukou” were more compliant with both protective and restrictive behaviours (p < 0.1), while demographic factors had no impact on non-young elites’ compliance behaviour.

We also found that the sense of social responsibility (p < 0.001) and trust in government (p < 0.001) were the major factor motivating young elites’ policy compliance. Non-young elites’ compliance with protective and restrictive behaviours tended to be affected by their previous experience of pandemic prevention (p < 0.05) and trust in government (p < 0.001). In contrast, young non-elites’ compliance with protective and restrictive behaviours were motivated by the sense of social responsibility (p < 0.05).

**Table 8:** The impacts of the proposed determinants on policy compliance using subsamples

*Variables*	*Young elites*		*Non-young elites*		*Young non-elites*	
	*Model 3*	*Model 4*	*Model 5*	*Model 6*	*Model 7*	*Model 8*
	*Protective behaviour*	*Restrictive behaviour*	*Protective behaviour*	*Restrictive behaviour*	*Protective behaviour*	*Restrictive behaviour*
Gender (Female)	-0.031*	-0.004	-0.008	0	0.04	0.026
Hukou (Rural)	0.057***	0.011	0.014	0.009	0.139***	0.076**
Political identity (CCP membership)	-0.062**	-0.045**	-0.031	0.028	0.126	0.009
The fear of being punished	-0.04**	0.022	-0.105**	-0.028	-0.111**	-0.115***
The fear of being infected and infecting family members and friends	-0.031	0.024	0.012	0.006	0.012	0.07
The previous experience of pandemic prevention	0.015	-0.018	0.099***	0.052*	0.025	0.085*
The sense of social responsibility	0.089***	0.048***	0.048	0.059*	0.144**	0.091*
Trust in government	0.098***	0.041***	0.141***	0.098***	0.059	0.067
Constant	3.674***	3.859***	3.538***	3.694***	3.531***	3.664***
Number of observations	1,932	1,932	861	861	329	329
Adj. R-squared	0.042	0.023	0.047	0.024	0.063	0.063

Note: *, ** and *** indicate p < 0.1, p < 0.05 and p < 0.01, respectively

## Discussion

### Discussion of key findings

This study used a sample comprising 3,122 Chinese individuals to explore and compare the compliance behaviour between college students and others during the COVID-19 pandemic. This study stressed that the sense of social responsibility and trust in government, instead of the fear of being punished, were the major drivers of young elites’ compliance behaviour during the COVID-19 pandemic.

Since the outbreak of COVID-19, a set of public health prevention and control policies have been proposed by the Chinese government to address challenges arising from the COVID-19 pandemic. Prior research has shown that a prolonged high level of self-discipline could easily lead to ‘behavioural fatigue’, which caused a gradual decline in risk perception and even resulted in negative health consequences [58]. However, our results revealed that individuals still maintained a high level of policy compliance after COVID-19 prevention and control policies had been implemented for a long period of time in China. Specifically, we found that of the various prevention and control measures, the highest level of policy compliance was found in relation to the provision of HCTC, followed by vaccination and NATC (Table 2).

We demonstrated that the deterrence motivation and protection motivation were not important predictors of individuals’ compliance behaviour (Tables 7 and 8). One unexpected finding was that risk perception was not associated with individuals’ compliance behaviour, which is in line with some prior research [38] while being inconsistent with other studies [48, 59]. Due to the implementation of strict prevention and control regulations, the number of infected cases and mortality rates remained low, which may weaken the impacts of risk perception in the long run. Another interesting finding was that the fear of punishment reduced citizens’ policy compliance, which is inconsistent with findings generated from prior work [5]. China’s authoritarian political system has the strength in ensuring that the containment measures were carried out in a strict and disciplined manner that were always associated with higher levels of policy compliance [7, 60, 61]. Nevertheless, the impacts of punitive measures may be weakened in the long term when individuals discovered that they were able to afford the costs of violating regulations and when a large number of individuals jointly refused to comply with the measures introduced.

We also found that the normative motivation (including the sense of social responsibility and trust in government) was the most important driver of individuals’ compliance behaviour (Tables 7 and 8), which is in line with prior studies [9, 32, 33, 47, 62–64]. Particularly, we found that young elites in China chose to follow the prevention and control measures because of their personal responsibility and trust in government, which is also consistent with previous conclusions [65–67]. These findings may be explained by the social and cultural factors [68] that Chinese citizens always placed collective interests above individual interests in a collectivist society in China [69].

Our study demonstrated that young elites who were male, had a rural “hukou”, and were not a member of the China Communist Party had a higher level of policy compliance than their counterparts (Table 3), which contradicts findings from previous studies [70–73]. In comparison with females, males faced a higher level of risks associated with the COVID-19 pandemic and had a larger mortality rate [74]; hence, males were more likely to comply with protective measures such as wearing a mask and vaccinating. Rural residents generally live in less dense environments where the risk of infection and the stringency of policy was smaller, which may explain why young elites with a rural “hukou” exhibited a higher level of policy compliance than their urban counterparts. Individuals who were a member of the China Communist Party have more flexibility to interpret policies and still have a limited political space within which to deviate from policy objectives [75]. Meanwhile, young elites with a party membership could also be influenced by values such as hedonism because of their limited life experience and poor discrimination capacity [76]. These facts may explain why young elites without a political party membership had a relatively higher level of policy compliance than those who were a member of the China Communist Party.

### Strengths and limitations

Since the outbreak of COVID-19, research into compliance behaviour and its motivating factors has neglected the population of young elites in China. This study contributed to the extant studies through demonstrating that young elites exhibited a relatively high level of compliance with COVID-19 prevention and control policies. Another important contribution of this study was that we demonstrated the normative motivation (rather than calculated motivation) was the most important driver of young elites’ compliance behaviour.

Nevertheless, some limitations of this study should be acknowledged. First, the degree of policy compliance was measured using subjective measures of which empirical social researchers remain skeptical [77]. Although the questionnaire was anonymous, some of the respondents inevitably were reluctant to disclose their non-compliance with policies and the reasons for their compliance. However, with the development of humanism, the analytical method of using subjective measures to explain subjective issues still offers valuable insights [78]. Second, while the study used survey data from three time periods, we simply combined the data as cross-sectional data, which are still not longitudinal and fail to provide causal estimates of the long-term effects of the proposed determinants. Third, this study used a small study sample, which may impair the generalizability of our study findings. We strongly recommend future studies to test the consistency of findings from this study using a larger sample from different administrative regions and countries. Finally, although our study found that the average level of compliance is high among young elites, it cannot be ignored that young people with low compliance are a minority but can be very unstable factors in maintaining the social order. Some studies also suggested that long-term strict epidemic prevention policies can increase negative emotions, such as anxiety and depression [17–19]. Even highly compliant individuals can cause mental health problems under long-term negative emotions, which potentially leading great challenges to social stability. Our research did not evaluate the relationship between negative emotions and compliance behaviour. This can be our further research direction. Since early December 2022, the Chinese government loosen the strict prevention and control policies, it suddenly relieved the pressure of people, including college students, for strictly implementation the epidemic prevention policies. Hence re-evaluate the previous strict policies and the later relaxed policies, which opened up the possibility of future research on citizens’ policy compliance behaviour in the aftermath of these policies.

## Conclusion

The successful containment of the pandemic depended not only on effective government policies but also on citizens’ compliance with relevant policies [30, 61]. This study found that the Chinese young elites generally had a high level of compliance with the prevention and control policies introduced by the government during the COVID-19 pandemic. The main reasons that young elites complied with the prevention and control measures were their sense of social responsibility and trust in government, rather than the fear of being infected or punished. Our research also found that young elites who were male, had a rural “hukou”, and were non-party members had higher policy compliance than their counterparts. Our findings provided new evidence for understanding factors impacting young elites’ compliance behaviour in the context of the pandemic. The results suggest that in future health crises, promoting young elites’ sense of social responsibility and building a relationship of trust with the young elites can help promote their policy.

## Data Availability

If you need us to provide data, please contact the correspondence author.

## Abbreviations

NATC: Nucleic acid test certificate
HCTC: Health code or travel card
CCP: China Communist Party.

## References

[1] Dong XS, Guo W, Zhao X. Implementation Mode and Policy Compliance in Crisis Recovery Management —— A Case Study of Citizen’s Deliberation as the Solution.Chinese Public Administration, 2011,107–112.

[2] Porumbescu G, Bellé, Nicola, Cucciniello M, Nasi G (2017). Translating policy transparency into policy understanding and policy support: evidence from a survey experiment. Public Adm.

[3] Fu CZ, Liao L, Huang WJ (2021). Behavioral implementation and compliance of anti-epidemic policy in the COVID-19 crisis. Int J Environ Res Public Health.

[4] Guan B, Bao G, Liu Q, Raymond RG. Two-way risk communication, public value consensus, and citizens’ policy compliance willingness about COVID-19: multilevel analysis based on nudge view. Adm Soc. 2021;53(5). 10.1177/0095399721990332

[5] Li Y, Su YD, Zhu CK, A Review of Research on Citizen Policy Compliance (2021). Based on the dual perspective of “Policy Situation” and “Behavior characteristics. J Public Adm.

[6] Chen S, Yang J, Yang W, Wang C, Brnighausen T. Covid-19 control in China during mass population movements at new year. The Lancet. 2020;395(10226). 10.1016/S0140-6736(20)30421-9 10.1016/S0140-6736(20)30421-9 PMC7159085 32105609

[7] Fu Y, Ma W, Wu J. Fostering voluntary compliance in the COVID-19 pandemic: an analytical framework of information disclosure. Am Rev Public Adm. 2020;50(6–7). 10.1177/0275074020942102

[8] Zhao T, Wu Z (2020). Citizen–state collaboration in combating COVID-19 in China: experiences and lessons from the perspective of co-production. Am Rev Public Adm.

[9] Zimmermann BM, Fiske A, McLennan S, et al. Motivations and limits for COVID-19 policy compliance in Germany and Switzerland. Int J health policy Manage. 2021;30. 10.34172/ijhpm.2021.30 10.34172/ijhpm.2021.30 PMC9808338 33949815

[10] Ferrante G, Baldissera S, Moghadam PF, et al. Surveillance of perceptions, knowledge, attitudes and behaviors of the italian adult population (18–69 years) during the 2009–2010 A/H1N1 influenza pandemic. Eur J Epidemiol. 2011;26(3). 10.1007/s10654-011-9576-3 10.1007/s10654-011-9576-3 21476080

[11] Springborn M, Chowell G, MacLachlan M, et al. Accounting for behavioral responses during a flu epidemic using home television viewing. BMC Infect Dis. 2015;15(1). 10.1186/s12879-014-0691-0 10.1186/s12879-014-0691-0 PMC4304633 25616673

[12] Wong LP, Sam I. “Temporal changes in psychobehavioral responses during the 2009 H1N1 influenza pandemic.” *Preventive medicine*, 2010,51,1: 92 – 3. 10.1016/j.ypmed.2010.04.010 10.1016/j.ypmed.2010.04.010 PMC7125894 20403375

[13] Chang B. An Analysis of the Discourse Right, Social Responsibility and Dilemma of Minority Intellectual Elite—Taking Inner Mongolia Mongolian Intellectual Elite as an Example.Journal of Northwestern Ethnic Studies, 2017(04):38–46.

[14] Zhao Q. An Analysis of the Predicament of the Minority Intellectuals in the Process of Modernization —— Taking the Uighur Intellectuals as an Example.Ningxia Social Sciences. 2017(04):38–46. 10.16486/j.cnki.62-1035/d.2017.04.005

[15] Sunstein CR (1996). Social norms and Social Roles. Columbia Law Rev.

[16] Tao SY, Cheng YL, Lu Y (2013). Handwashing behaviour among chinese adults: a cross-sectional study in five provinces. Public Health.

[17] Cam HH, Top FU, Ayyildiz TK. Impact of the COVID-19 pandemic on mental health and health-related quality of life among university students in Turkey. Curr Psychol. 2021;3. 10.1007/s12144-021-01674-y 10.1007/s12144-021-01674-y PMC8011049 33814870

[18] Gao W, Ping S, Liu X. Gender differences in depression, anxiety, and stress among college students: a longitudinal study from China. J Affect Disord. 2019;263. 10.1016/j.jad.2019.11.121 10.1016/j.jad.2019.11.121 31818792

[19] Cao WJ, Fang ZW, Hou GQ (2020). The psychological impact of the COVID-19 epidemic on college students in China. Psychiatry Res.

[20] Sultana M, Mahumud RA, Sarker AR, Hossain SM. Hand hygiene knowledge and practice among university students: evidence from Private Universities of Bangladesh.Risk Management and Healthcare Policy,2016(Issue 1). 10.2147/RMHP.S98311 10.2147/RMHP.S98311 PMC4758791 26929673

[21] Zhong BL, Luo W, Li HM (2020). Knowledge, attitudes, and practices towards COVID-19 among chinese residents during the rapid rise period of the COVID-19 outbreak: a quick online cross-sectional survey. Int J Biol Sci.

[22] Zhu XF. Transition of Policy-Making and Elite Advantage.Sociological Studies, 2008(02):69–93 + 244.

[23] Pedersen MJ, Favero N (2020). Social Distancing during the COVID pandemic: who are the Present and Future non-compliers?. Public Adm Rev.

[24] Burby RJ, Paterson RG (1993). Improving compliance with State Environmental Regulations. J Policy Anal Manag.

[25] Winter SC, May PJ (2001). Motivation for compliance with Environmental Regulations. J Policy Anal Manage.

[26] Egüez A. Compliance with the EU Waste Hierarchy: It is a matter of stringency, enforcement and time! *CERE Working Papers*, 2020. 10.1016/j.jenvman.2020.111672 10.1016/j.jenvman.2020.111672 33309110

[27] Skinner WF, Fream AM (1997). A Social Learning Theory Analysis of Computer Crime among College Students. J Res Crime Delinquency.

[28] Li H, Sarathy R, Zhang J, Luo X. Exploring the effects of organizational justice, personal ethics and sanction on internet use policy compliance.Information Systems Journal,2014,24(6).

[29] Floyd D, Prentice-Dunn S, Rogers RW. A Meta‐Analysis of Research on Protection Motivation Theory. J Appl Soc Psychol. 2000;30(2). 10.1111/j.1559-1816.2000.tb02323.x

[30] de Zwart O, Veldhuijzen IK, Elam G (2007). Avian influenza risk perception, Europe and Asia. Emerg Infect Dis.

[31] Leppin A, Aro AR (2009). Risk perceptions related to SARS and Avian Influenza: theoretical foundations of current empirical research. Int J Behav Med.

[32] Murphy K, Williamson H, Sargeant E, et al. Why people comply with COVID-19 social distancing restrictions: self-interest or duty? Australian and New Zealand Journal of Criminology. 2020;53(1). 10.1177/0004865820954484

[33] Saechang O, Yu JX, Li Y (2021). Public trust and policy compliance during the covid-19 pandemic: the role of professional trust. Healthcare.

[34] Park JH, Cheong HK, Son DY, Kim SU, Ha CM (2010). Perceptions and behaviors related to hand hygiene for the prevention of H1N1 influenza transmission among korean university students during the peak pandemic period. BMC Infect Dis.

[35] Helene ACM, de Voeten; Onno Z, Irene K, Veldhuijzen (2009). Cicely Yuen; Xinyi Jiang; Gillian Elam; Thomas Abraham; Johannes Brug. Sources of information and health beliefs related to SARS and avian influenza among chinese communities in the United Kingdom and the Netherlands, compared to the general population in these countries. Int J Behav Med.

[36] Jiang XY, Elam G, Yuen C (2009). The perceived threat of SARS and its impact on precautionary actions and adverse consequences: a qualitative study among chinese communities in the United Kingdom and the Netherlands. Int J Behav Med.

[37] Ren W, Zhu XW, Hu Y (2021). Risk perception and Epidemic Prevention Behavior: a comparative study of the multiple functions of Social Media and authoritative media in the context of COVID-19 epidemic. Chin J Journalism Communication.

[38] Zhou LY, Liu TF. Risk Perception and Preventive Behavior of COVID-19 from the Perspective of Information.Fudan Public Administration Review, 2021(01):123–147.

[39] Wang L, Wang YM (2013). Research on emergency decision method of dynamic reference point based on prospect theory. Chin J Manage Sci.

[40] Fan ZP, Liu Y, Shen RJ (2012). Risk decision method of emergency response based on prospect theory. Syst Engineering-Theory Pract.

[41] Moon MJ (2020). Fighting against covid with agility, transparency, and participation: wicked policy problems and new governance challenges. Public Adm Rev.

[42] Amirkhanyan AA, Meier KJ, O’Toole LJ. Managing in the Regulatory Thicket: Regulation Legitimacy and Expertise. Public Adm Rev. 2017;77(3). 10.1111/puar.12591

[43] Tobin I, Wonhyuk C, Greg P et al. Internet, Trust in Government, and Citizen Compliance.Journal of Public Administration Research & Theory, 2014(3):741–763. 10.1093/jopart/mus037

[44] Raymond L, Cason TN (2011). Can affirmative motivations improve compliance in Emissions Trading. Programs? Policy Studies Journal.

[45] Yazdanmehr A, Wang J (2016). Employees’ information security policy compliance: a norm activation perspective. Decis Support Syst.

[46] Janmaimool P, Khajohnmanee S. Enhancing university students’ global citizenship, public mindedness, and moral quotient for promoting sense of environmental responsibility and pro-environmental behaviours.Environment, Development and Sustainability,2020,22(2).

[47] Ning LW, Hao YH, Liu Z (2021). Major influencing factors of high public compliance behavior in China during regular prevention and control of COVID-19 epidemic: a random forest model analysis. Chin J Public Health.

[48] Chong YY, Chien WT, Cheng HY (2020). The role of illness perceptions, coping, and self-efficacy on adherence to precautionary measures for COVID-19. Int J Environ Res Public Health.

[49] Brekke KA, Kipperberg G, Nyborg K (2010). Social Interaction in responsibility ascription: the Case of Household Recycling. Land Econ.

[50] Wang WQ, Lai T (2021). Urban residents’ response to the dilemma of community governance in the context of public crisis —— taking Shanghai residents’ participation i n the prevention and control of COVID-19 epidemic as an example. J Hohai University(Philosophy Social Sciences).

[51] Bücker J, Rosa AR, Czepielewski LS. Impact of the COVID-19 pandemic on mental health among local residents in South of Brazil: during pandemic times, youth sleep matters. Trends Psychiatry Psychother. 2022;44. 10.47626/2237-6089-2021-0225 10.47626/2237-6089-2021-0225 PMC9995138 33974774

[52] Gustavsson J, Beckman L. Compliance to recommendations and Mental Health Consequences among Elderly in Sweden during the initial phase of the COVID-19 Pandemic—A Cross Sectional Online Survey. Int J Environ Res Public Health. 2020;17(15). 10.3390/ijerph17155380 10.3390/ijerph17155380 PMC7432611 32722624

[53] Guzek D, Skolmowska D, Gbska D. Analysis of gender-dependent personal protective behaviors in a National Sample: polish adolescents’ COVID-19 experience (PLACE-19) study. Int J Environ Res Public Health. 2020;17(16). 10.3390/ijerph17165770 10.3390/ijerph17165770 PMC7459707 32785004

[54] Yu Y. On the position and role of intellectuals from the perspective of post-modern social theory.Social Science Research, 2007(03):101–105.

[55] Liu Y, Gu Z, Xia S, et al. What are the underlying transmission patterns of COVID-19 outbreak? – an age-specific social contact characterization. EClinicalMedicine. 2020;22. 10.1016/j.eclinm.2020.100354 10.1016/j.eclinm.2020.100354 PMC7165295 32313879

[56] Nussbaumer-Streit B, Mayr V, Dobrescu AI et al. Quarantine alone or in combination with other public health measures to control COVID-19: a rapid review.Cochrane database of systematic reviews (Online), 2020, 4(2). 10.1002/14651858.CD013574 PMC7141753 32267544

[57] World Health Organization (WHO). Orientation programme on adolescent health for health care providers.Geneva World Health Organization, 2006. https://apps.who.int/iris/bitstream/handle/10665/42868/924459126x_Guide_rus.pdf

[58] Bults M, Beaujean DJMA, de Zwart OD (2011). Perceived risk, anxiety, and behavioural responses of the general public during the early phase of the Influenza A (H1N1) pandemic in the Netherlands: results of three consecutive online surveys. BMC Public Health.

[59] Weerd VDW, Daniëlle RT, Desirée JB (2011). Monitoring the level of government trust, risk perception and intention of the general public to adopt protective measures during the influenza a (h1n1) pandemic in the Netherlands. BMC Public Health.

[60] Kupferschmidt K, Cohen J (2020). Can China’s COVID-19 strategy work elsewhere?. Science.

[61] Cheng YD, Yu J, Shen Y, Huang B (2020). Coproducing responses to COVID with community-based organizations: lessons from Zhejiang province, China. Public Adm Rev.

[62] Fang M, Zhang H (2021). How can crisis intervention repair government trust? ——the moderating effect of risk communication and community support. J Public Adm.

[63] Tyler TR. Why people obey the Law[M]. Yale University Press; 2006.

[64] Vu VT (2021). Public Trust in Government and Compliance with Policy during COVID-19 pandemic: empirical evidence from Vietnam. Public Organ Rev.

[65] Deurenberg-Yap M, Foo LL, Low YY et al. The Singaporean response to the SARS outbreak: knowledge sufficiency versus public trust.Health Promotion International, 2005(4). 10.1093/heapro/dai010 10.1093/heapro/dai010 PMC7108623 15964886

[66] Gilles I, Bangerter A, Clémence A et al. Trust in medical organizations predicts pandemic (H1N1) 2009 vaccination behavior and perceived efficacy of protection measures in the Swiss public.European journal of epidemiology, 2011(3). 10.1007/s10654-011-9577-2 10.1007/s10654-011-9577-2 21476079

[67] Paek HJ, Hilyard K, Freimuth VS (2008). Public support for government actions during a flu pandemic: lessons learned from a statewide survey. Health Promot Pract.

[68] Cialdini RB, Goldstein NJ (2004). Social Influence: compliance and conformity. Ann Rev Psychol.

[69] Cialdini RB, Wosinska W, Barrett DW (1999). Compliance with a request in two cultures: the Differential influence of Social Proof and Commitment/ consistency on Collectivists and Individualists. Personal Soc Psychol Bull.

[70] Gibson MJ, Hartman TK, Levita L et al. Capability, opportunity, and motivation to enact hygienic practices in the early stages of the COVID-19 outbreak in the United Kingdom.British journal of health psychology,2020,25(4). 10.1111/bjhp.12426 10.1111/bjhp.12426 PMC7276910 32415918

[71] Cauteren DV, Vaux S, Valk HD (2012). Burden of influenza, healthcare seeking behaviour and hygiene measures during the A(H1N1)2009 pandemic in France: a population based study. BMC Public Health.

[72] Yi WG, Fang F, Cheng XM (2020). Observation and thinking on the effectiveness of community governance in the prevention and control of major epidemic situations. Jiangxi Social Sciences.

[73] Zhong Y. Do Chinese People Trust their local government, and why? Probl Post-Communism. 2014;61(3). 10.2753/PPC1075-8216610303

[74] Rasmussen SA, Smulian JC, Lednicky JA, et al. Coronavirus Disease 2019 (COVID-19) and pregnancy: what obstetricians need to know. Am J Obstet Gynecol. 2020;0002937820301976. 10.1016/j.ajog.2020.02.017 10.1016/j.ajog.2020.02.017 PMC7093856 32105680

[75] Yang SH. Family Politics and the selection, Role Orientation and Elite replacement of rural grassroots political elites —— An Analytical Framework. Sociol Stud. 2000;03101–8. 10.19934/j.cnki.shxyj.2000.03.008

[76] Wang LH (2012). Ways and Methods of innovating party member’s Ideal and Belief Education for College Students —— based on the investigation and analysis of 18 universities in China. J Natl Acad Educ Adm.

[77] Janmaat JG, Subjective Inequality (2013). A review of International Comparative Studies on people’s views about Inequality. Eur J Sociol.

[78] Hu AN (2019). Explaining one subjective variable with another: a methodological clarification. Chin J Sociol.

